# Complete resolution of otogenic cerebellar abscess with conservative approach: Two case reports

**DOI:** 10.1016/j.amsu.2022.104001

**Published:** 2022-06-16

**Authors:** Riva Satya Radiansyah, Paulus Sugianto, Cindy Cecilia

**Affiliations:** Department of Neurology, Faculty of Medicine, Universitas Airlangga – Dr. Soetomo General Academic Hospital, Surabaya, Indonesia

**Keywords:** Cerebellar abscess, Conservative management, Otitis media

## Abstract

**Background:**

Cerebellar abscess is rare, and these two case reports are examined to consider alternative therapy.

**Case presentation:**

We present two cases of patients with cerebellar abscess. In both cases, patients had the same initial symptoms of fever and central vertigo, with otitis media as the source of infection. However, one patient had generalized onset of tonic-clonic seizure. Both patients were given the same regimen of antibiotics for six weeks and then, evaluated on a clinical and radiological basis via computed tomography (CT) and magnetic resonance imaging (MRI). Sixth weeks after treatment was initiated, No. abscesses were detected in either patient; clinically, there were no complaints or neurological deficits.

**Discussion:**

There are several therapeutic management options in cases of cerebellar abscess. With conservative management (e.g., administering symptomatic drugs and antibiotics), patients can recover completely.

**Conclusion:**

Management of patients with cerebellar abscess is very challenging, but adequate therapy and appropriate prevention of complications can help reduce morbidity and mortality.

## Highlights

• Otogenic infection is a major cause of cerebellar abscess.
• Conservative management can be used to treat cerebellar abscess.
• Complete resolution of otogenic cerebellar abscess without neurological deficits can be achieved with antibiotic treatment.

## 1 Introduction

Brain abscess is a focal suppurative condition in the parenchyma of the brain [1,2]. The cerebellum is one of the most common intracranial locations in brain abscess due to otitis media and mastoiditis [1,3]. One or more symptoms, such as fever, headache, decreased of consciousness, or focal neurological symptoms, including seizures, balance difficulties, dysphagia, or focal sensorimotor impairments, are common clinical characteristics [4]. Brain abscess as a universal health problem with high morbidity and mortality rates which also causes a major widespread public health problem in the worldwide [5]. The development of non-invasive radiological diagnostic tools, antibiotics that transcend the blood-brain barrier and enter the abscess itself, and minimally invasive surgical procedures have altered the diagnosis and management of cerebellar abscess in recent decades [1]. Therefore, we are interested in reporting two cases of cerebellar abscess that were complete resolution with conservative management. This case report has been reported in line with the SCARE Criteria [6].

## 2 Case presentation

### 2.1 Case 1

A 14-year-old girl presented with generalized onset of tonic-clonic seizures with a duration of approximately 1 minute. The seizures occurred twice; the second seizure occurred 1 h after the first seizure and had the same form. Two days earlier, the patient had experienced vertigo and fever. Vertigo was felt continuously and did not improve with a change in position. Nausea and vomiting were also present. A week earlier, she had experienced left ear pain and yellow discharge. She had never experienced a similar illness. On physical examination, she was conscious; normal vital signs with neurological deficits were diagnosed as left abducens palsy, left facial palsy (peripheral type), bidirectional horizontal nystagmus, and slight left hemiparesis.

**Fig. 1:**
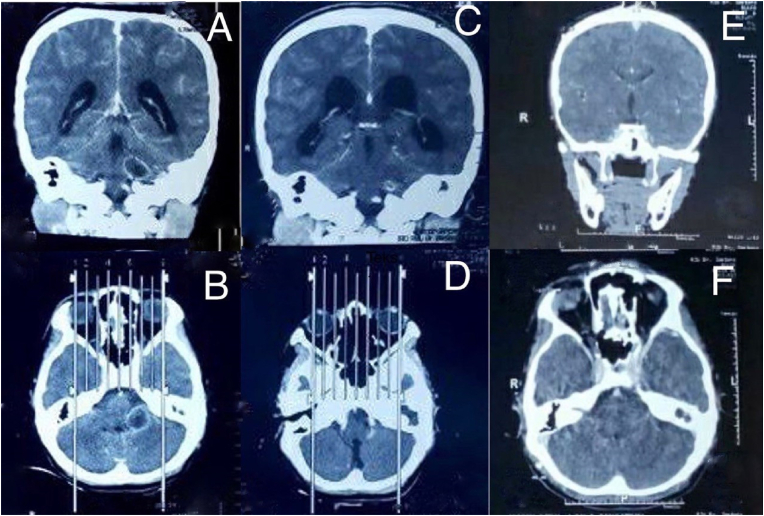
Case 1. A, B Initial CT head scan with contrast. C, D CT head scan at the third week of antibiotic administration. E, F CT head scan at the sixth week of antibiotic administration.

**Fig. 2:**
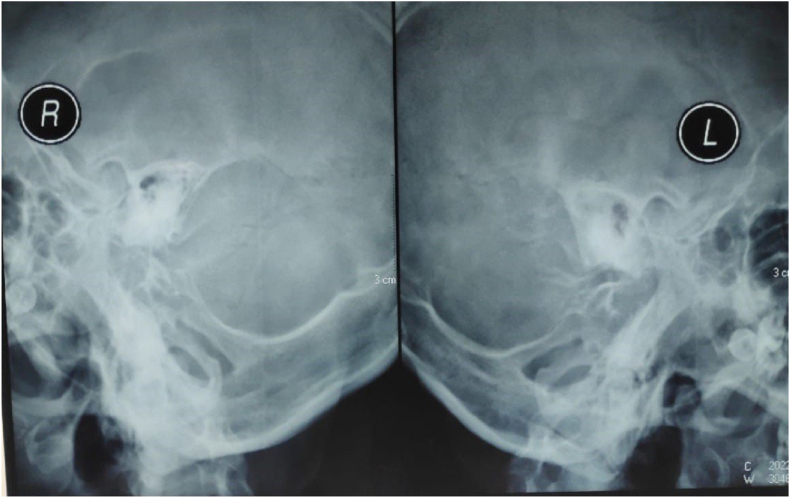
Schuller's photo case 1.

Abnormal results were initially obtained in the emergency room, namely leukocytosis of 14.800 μL and slight hypernatremia of 147 mmol/L. A computed tomography (CT) head scan with contrast showed a left cerebellar abscess (dimensions 2 × 1.8 × 1.4 cm) accompanied by communicating hydrocephalus (Fig. 1 A, B). She also performed Schuller's photo to find the infection source; right and left chronic sclerotic-type osteomastoiditis was identified (Fig. 2).

The patient and family declined surgical intervention for diagnosis or treatment. During hospitalization, she was given the antibiotic ceftriaxone twice daily (2 g every 12 hours) and 500 mg of metronidazole every 6 hours intravenously. To prevent seizure recurrence, she was given phenytoin intravenously (100 mg every 8 hours); for her ear infection, she was given 3% hydrogen-peroxide ear drops and fluoroquinolone ear drops every 8 hours. After four days of treatment, she underwent an electroencephalogram, results showed background slow activity and continuous slow activity with the possibility of generalized epileptogenic.

After three weeks of antibiotics, the CT head scan showed that the cerebellar abscess had shrunk to a size of 0.8 × 0.9 × 0.8 cm (Fig. 1C, D). Clinically, she had no complaints of seizure or dizziness and did not have any neurological deficits. In the sixth week, no cerebellar abscess was found in the CT head scan (Fig. 1 E, F). She was discharged from hospital and antibiotics were discontinued.

### 2.2 Case 2

An immunocompetent 45-years-old male, presented with low-grade fever, severe central vertigo, progressive cerebellar ataxia, and other cerebellar syndromes which had been present for two years and became prominent four months before hospital admission. He had a three-year history of chronic otitis media, which had been treated by antibiotics several times and by aural toileting several times.

Physical examinations showed stable vital signs, subfebrile temperature, and full consciousness with a Glasgow coma scale score of 15. No nuchal rigidity, papilledema, or other signs of increased intracranial pressure were observed. Primary neurological deficit was central vertigo with left and right cerebral disfunction. Otology and mastoid examination showed chronic otitis media without acute infection, discharge, or tenderness. Laboratory investigations showed normal values (i.e., negative for blood and ear cultures); however, an audiogram showed mixed hearing loss for the right ear. Chest, paranasal, and skull X-rays were also normal.

**Fig. 3:**
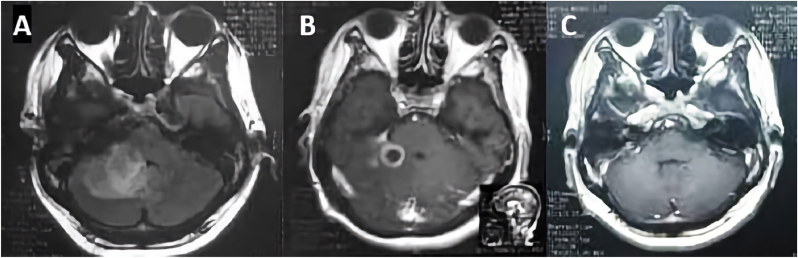
Case 2. A Initial head MRI with contrast. B CT head scan at the fourth week of antibiotic administration. C MRI at the sixth week of antibiotic administration.

The head magnetic resonance imaging (MRI) with contrast showed one larger and one smaller abscess in the right cerebellar lobe with respective dimensions of ±1.8 × 1.7 × 2.2 cm and ±1.7 × 1.5 × 2 cm (Fig. 3A). Both were hypointense in T1 and hyperintense in T2. There were no signs of brainstem compression or increased intracranial pressure. The patient was diagnosed with multiple otogenic cerebellar abscesses.

The patient declined surgical intervention for diagnosis and treatment. He was treated with intravenous ceftriaxone (2 g every 12 hours) and metronidazole (500 mg every 6 h); he was also given symptomatic treatment for central vertigo with betahistine mesylate, diphenhydramine and flunarizine. These treatments were administered for one month. The abscesses shrank into a single nodule (±0.6 × 0.6 × 0.9 cm) after four weeks of antibiotic administration, and the patient was discharged with minimal neurological deficit (Fig. 3B). Symptoms of vertigo and cerebellar syndrome decreased significantly, and the patient regained the ability. Antibiotics were continued for another 2 weeks and MRI was performed (Fig. 3C). The abscess had resolved completely.

## 3 Discussion

In Western countries, otogenic brain abscess accounts for 1%–2% of space-occupying processes in the brain, whereas, in developing countries, it accounts for 8% [7]. The largest risk factor is the transmission of diseases from nearby areas (50% of cases) after otitis, mastoiditis, sinusitis, meningitis, neurosurgery, or traumatic brain injury or through hematogenous spreading [8]. Once the blood–brain barrier has been penetrated, the brain is extremely vulnerable to bacterial infection. Cerebellar abscess is the second-most-common complication after meningitis, which is caused by otitis media [5,7,8]. In a typical pyogenic abscess, the outer wall made up of collagen, granulation tissue, macrophages, and gliosis which surrounds the central necrotic tissue [3].

The earliest stage of brain abscess is early cerebritis, which can cause a perivascular inflammatory response around the necrotic center, accompanied by edema. Subsequently, the necrotic center grows to its maximum size, and a capsule is formed through fibroblast accumulation and new vascularization. The capsule is thickened by a large amount of reactive collagen, yet inflammation and edema extend beyond it [3]. Around the abscess capsule, astrogliosis and cerebral edema can be detected. Gliosis can last for months or years following therapy, and it's a major risk factor for the development of post-infectious epilepsy [7]. However, in the two cases presented here, the abscesses were completely resolved without sequelae.

Brain abscess is commonly typified by the following symptoms: 82.5% headache, 75% altered levels of consciousness, and 66.9% papilledema [8]. Neurological symptoms vary depending on where the abscess is located and can be modest or severe. Patients with brainstem or cerebellar abscesses may present with cranial nerve palsies, gait disturbances, headaches, or altered levels of consciousness due to hydrocephalus [9]. Seizures affect up 25% of patients. Brain scans should be performed for all patients with suspected brain abscess. CT head scans with contrast provide a quick way to detect abscess size, number, and localization [2,10].

Neurosurgical drainage is the preferred treatment for a brain abscess [11]. Twenty years ago, total resection was often recommended but now has a limited role due to medical advances and minimally invasive neurosurgery. Neurosurgical intervention has been suggested for abscesses with a diameter greater than 2.5 cm; however, there is a lack of evidence from comparison research, thus this size cannot be considered a definitive indication for aspiration. Regardless of size, neurosurgical intervention may be required for patients whose abscess have caused a shift in the brain, resulting in brain herniation [3].

Broad-spectrum antibiotics that can cross the blood–brain barrier and blood–cerebrospinal fluid barrier in sufficient concentrations should be used as the first line of treatment. Empirical antibiotics should include coverage for anaerobic organism, such as third-generation cephalosporins and metronidazole, as well as vancomycin if the patient has a history of penetrating trauma or recent neurosurgical procedures [3,10]. In patients with bacterial brain abscess, intravenous antibiotic therapy has traditionally lasted six to eight weeks. Delays in initiating antibiotics therapy can lead to poor results, as demonstrated by a retrospective study in which the median gap between diagnosis and initiation of antibiotic therapy was two days [3]. Metronidazole can easily penetrate an abscess; the intralesional dose has been found to be 40 mg/ml. It is highly effective against many anaerobes but ineffective against aerobic organisms. Based on these characteristics, many experts recommend this medication for the majority of patients with cerebral abscess [4].

In the two cases presented above, the patients began to improve clinically after receiving antibiotics intravenously, with brain imaging showing a reduction in abscess size. Both patients responded well to medical management. Antibiotic therapy was continued for six weeks, and post-treatment brain scans revealed abscess resolution.

## 4 Conclusion

Conservative management of cerebellar abscess is unusual and very challenging. In Indonesia, the success rate for handling this cases is still low. In these two cases, both patients were immunocompetent and they refused to undergo surgical intervention. Regardless, it was demonstrated that an otogenic cerebellar abscess could respond to antibiotic treatment and symptomatic therapy. The abscesses were completely resolve and the patients recovered without neurologic sequelae. In light of this information, future research should examine conservative management as the main option to treat cerebellar abscess.

## Ethical approval

Exempting ethical approval.

## Source of funding

None.

## Declaration of competing interest

None.

## Consent

Written informed consent was obtained from the adult patient and the parents of the 14-year old child for publication of this case report and accompanying images. A copy of the written consent is available for review by the Editor-in Chief of this journal on request.

## Author contribution

All authors contributed equally to this work including writing and critical revision.

## Registration of research studies

None.

## Guarantor

Riva Satya Radiansyah, Paulus Sugianto, Cindy Cecilia.

## Provenance and peer review

Not commissioned, externally peer reviewed.

## References

[1] Brook I. (2017). Microbiology and treatment of brain abscess. J. Clin. Neurosci..

[2] Utama A.S., Parengrengi M.A. (2022). Giant brain abscess in A pediatric patient with congenital heart disease: a case report. J. Heal. Sci. Med. Res..

[3] Brouwer M.C., Tunkel A.R., McKhann G.M., Van de Beek D. (2014). Brain abscess. N. Engl. J. Med..

[4] Alvis-Miranda H., Castellar-Leones S., Elzain M., Moscote-Salazar L. (2013). Brain abscess: current management. J. Neurosci. Rural Pract..

[5] Menon S., Bharadwaj R., Chowdhary A., Kaundinya D.V., Palande D.A. (2008). Current epidemiology of intracranial abscesses: a prospective 5 year study. J. Med. Microbiol..

[6] Agha R.A., Franchi T., Sohrabi C. (2020). The SCARE 2020 guideline: updating consensus surgical CAse REport (SCARE) guidelines. Int. J. Surg..

[7] Feraco P., Donner D., Gagliardo C. (2020). Cerebral abscesses imaging: a practical approach. J. Popul. Ther. Clin. Pharmacol..

[8] Brouwer M.C., Coutinho J.M., Van De Beek D. (2014). Clinical characteristics and outcome of brain abscess :Systematic review and meta-analysis. Neurology.

[9] Shaw M.D., Russell J.A. (1975). Cerebellar abscess. J. Neurol. Neurosurg. Psychiatry.

[10] Sugianto P., Sutantoyo F.F. (2021). Skipped multilevel lesion as an atypical tuberculous spondylitis mimicking spinal metastasis: a case report. Neurol. Asia.

[11] Viswanatha B., Nsaeeruddin K. (2012). Conservative management of otogenic brain abscess with surgical management of attico antral ear disease: a review. Indian J. Otolaryngol. Head Neck Surg..

